# Review of Efficacy and Safety of Duloxetine 40 to 60 mg Once Daily in Patients with Diabetic Peripheral Neuropathic Pain

**DOI:** 10.1155/2012/898347

**Published:** 2012-08-29

**Authors:** Vladimir Skljarevski, Elijah P. Frakes, Doron Sagman, Sarah Lipsius, Alexandra N. Heinloth, Héctor J. Dueñas Tentori

**Affiliations:** ^1^Department of Neuroscience, Eli Lilly and Company, Indianapolis, IN 46285, USA; ^2^Department of Neuroscience, Eli Lilly Canada, Toronto, Canada ON M1N 2E8; ^3^Department of Strategic Resourcing, PharmaNet/i3, Ann Arbor, MI 48108, USA; ^4^Department of Neuroscience, Eli Lilly de México, 03900 México, Mexico

## Abstract

We summarize efficacy and safety findings from 4 double-blind, placebo-controlled, 12-week studies and 1 open-label, uncontrolled, 34-week maintenance-of-effect (MOE) study that examine duloxetine 40 and 60 mg once daily (QD) in patients with diabetic peripheral neuropathic pain (DPNP). In all placebo-controlled studies, duloxetine showed significantly (*P* ≤ .01) greater reduction in pain severity (weekly mean of 24-hour average pain severity ratings, primary outcome measure) compared with placebo. In all placebo-controlled studies, duloxetine showed significantly (*P* ≤ .05) greater improvement on brief pain inventory-Interference ratings. Patient global impression of improvement ratings were superior to placebo (*P* ≤ .01) for duloxetine patients in all placebo-controlled studies. Response rates (based on 30% pain reduction) ranged from 57% to 68% for duloxetine and from 35% to 47% for placebo and were statistically significantly different (*P* ≤ .01) between treatment groups in 3 out of 4 studies. The open-label study showed maintenance of analgesic effect of duloxetine in DPNP. In the duloxetine groups, 4.3% to 14.9% of patients discontinued because of adverse events (placebo groups: 2.6% to 7.4%). Most commonly reported treatment-emergent adverse events were nausea, somnolence, and headache. Duloxetine 40 and 60 mg QD was efficacious and well tolerated in the management of DPNP.

## 1. Introduction

Worldwide, the number of people with diabetes mellitus (DM) has more than doubled over the last 3 decades [1]. In 2008, the prevalence of DM in adults was 9.8% in men and 9.2% in women, globally [2]. Asian countries report a rapidly increasing prevalence of DM [3]. Symmetrical, sensorimotor diabetic peripheral neuropathy (DPN) is a common complication of DM. A significant number of patients with DPN suffer from diabetic peripheral neuropathic pain (DPNP) Among adults with DM, the estimates of prevalence of DPN range from 26% to 47% [4]. In a recent literature review, Sadosky and colleagues estimated the overall prevalence of DPNP in the population of patients with DM to be 15% [5].

Duloxetine is a selective serotonin and noradrenaline reuptake inhibitor (SNRI) [6, 7]. It possesses an analgesic effect [8], which is believed to be related to modulation of descending inhibitory pain pathways in the brain and spinal cord [9–11].

As of September 2011, duloxetine has been approved for the management of DPNP in 62 countries worldwide, including many Asian countries, most recently Japan. Doses of less than 40 mg/day have not demonstrated efficacy in the management of DPNP, and there is no evidence that 120 mg/day confers a significant additional benefit over 60 mg/day [12]. Therefore, in this report, we summarize efficacy and safety findings from clinical studies examining duloxetine 40 mg QD and 60 mg QD in patients with DPNP.

## 2. Materials and Methods 

Presented here are the efficacy and safety results from clinical trials that examined the effects of fixed doses of duloxetine 40 mg QD and 60 mg QD in patients with DPNP (Table 1). For all studies, the protocols were reviewed and approved by the appropriate institutional review boards before study initiation. All patients provided written informed consent before undergoing any study procedures, and all studies were conducted according to good clinical practice guidelines.

### 2.1. Studies 1 to 3, Double Blind, Placebo Controlled

Studies 1 to 3 were parallel, multicenter, placebo-controlled, fixed-dose clinical trials with 12-week, double-blind treatment phases primarily conducted in North and South America and Europe [13–15]. The primary objective of all 3 studies was to assess the efficacy of duloxetine on the reduction of pain severity, as compared with placebo, in patients with DPNP; severity was determined according to weekly mean of the 24-hour Average Pain Severity ratings. Enrolled were patients of either gender, ≥18 years of age, with DPNP present for at least 6 months. At baseline, patients were required to have a score of ≥3 on the Michigan Neuropathy Screening Instrument (MNSI) and a rating of ≥4 on the weekly mean of 24-hour Average Pain severity, which uses an 11-point (0–10) numerical scale. Eligible patients (study 1: *N* = 457; study 2: *N* = 334; study 3: *N* = 348) were randomized in study 1 in a 1 : 1 : 1 : 1 ratio to duloxetine 20 mg QD, 60 mg QD, 60 mg twice daily (BID), or placebo and in studies 2 and 3 in a 1 : 1 : 1 ratio to duloxetine 60 mg QD, 60 mg BID, or placebo [13–15]. 

### 2.2. Study 4, Double Blind, Placebo Controlled

Study 4 was a parallel, multicenter, placebo-controlled, fixed-dose clinical trial with a 12-week double-blind treatment phase conducted in Japan [16, 18]. The primary objective of the study was to assess the efficacy and safety of duloxetine 40 mg QD or 60 mg QD (combined group) compared with placebo on reduction of pain severity, assessed using the weekly mean of the 24-hour Average Pain severity ratings, in patients with DPNP. Enrolled were patients of either gender, aged ≥20 and <80 years. Patients had to have daily pain for ≥6 months, no peripheral neuropathy other than DPN, a decrease or loss of bilateral Achilles tendon reflex, bilateral hypopallesthesia in the area of medial malleolus, and a 24-hour Average Pain severity rating of ≥4 on a 0-to-10 scale. Eligible patients (*N* = 338) were randomized in a 2 : 1 : 1 ratio to placebo (*n* = 167), duloxetine 40 mg QD (*n* = 85), or duloxetine 60 mg QD (*n* = 86) at the beginning of the double-blind treatment phase [16, 18].

### 2.3. Study 5, Open Label, Uncontrolled

An open-label, multicenter, uncontrolled clinical trial with an 8-week treatment phase which was followed by a 26-week maintenance-of-effect (MOE) phase was conducted in South America and Europe [17]. The primary objective of this study was to evaluate whether an effect of duloxetine 60 mg QD was maintained over 6 months of therapy in patients with DPNP as measured by change on the 24-hour Average Pain severity rating. Only patients reporting a ≥30% pain reduction after the initial 8 weeks of therapy (responders) were included in the primary analysis. Eligible patients (*N* = 216, fulfilling the same entry criteria as described for studies 1 to 3, received duloxetine 30 mg QD for 1 week, followed by duloxetine 60 mg QD for 7 weeks. Responders (*n* = 115) continued to receive duloxetine 60 mg QD for an additional 26 weeks [17].

### 2.4. Efficacy Measures

The primary efficacy measure for studies 1 to 4 was mean change from baseline to 12 weeks in the weekly mean of 24-hour average pain severity ratings (11-point [0–10] numerical scale), from here on called pain severity rating. The pain severity ratings were computed from daily diary entries by the patients [13–15]. For study 5, mean change in 24-hour Average Pain severity rating over the 26-week maintenance phase was the primary efficacy measure [17].

In studies 1 to 4, secondary efficacy measures included clinically significant response rate (based on 30% and 50% pain reduction at 12 weeks), mean change from baseline to 12 weeks in Brief Pain Inventory (BPI)-Interference and Patient Global Impression (PGI)-Improvement at 12 weeks; in addition, in studies 1 to 3, mean change from baseline to 12 weeks in the Short Form 36 (SF-36) and EuroQol 5D Health Questionnaire (EQ-5D) was also examined [13–15, 20].

### 2.5. Analyses Methods for Previously Reported Primary and Secondary Efficacy Measures

All analyses were conducted on an intent-to-treat basis. All randomized patients were included in the safety analyses and all randomized patients with ≥1 postbaseline assessment were included in efficacy analyses.

In studies 1 to 4, a mixed-model repeated measures analysis was used for changes from baseline in pain severity ratings, BPI-Interference, SF-36 (not collected in study 4), and EQ-5D (not collected in study 4). The type III sum of squares was used to test between-treatment-group differences [13–16, 20].

In study 5, the null hypothesis was tested with a non-inferiority test evaluating a 1-sided 97.5% confidence interval (CI) of change from baseline to endpoint on the 24-hour Average Pain severity rating. A non-inferiority margin of 1.5 points on the 24-hour Average Pain Severity rating was set *a priori *as the upper bound of the 1-sided 97.5% CI—if this margin was not reached, the null hypothesis was rejected at a significance level of 0.025. In this analysis, baseline was defined as the observation at week 8, and endpoint was defined as the last nonmissing observation during the 26-week maintenance phase with duloxetine 60 mg QD [17].

Due to extensive similarities in study designs and patient populations, it was possible to pool data from studies 1 to 3. The pooled data set was stratified by age (<65 years of age versus ≥65 years of age) to explore potential age-dependent effects in efficacy and safety parameters [21]. To compare the probability of response for both age groups, a logistic repeated measures model was used [21]. Additionally, the pooled data were used to evaluate changes from baseline to 12-week endpoint in SF-36, BPI-Interference, and EQ-5D [22]. An ANCOVA model with main effects of treatment and study was used including baseline as the covariate. Treatment-by-study interaction was included at a 0.10 significance level. No adjustments were made for multiple comparisons [22].

### 2.6. Additional Analyses Used in the Current Review

*Post hoc*, we calculated number needed to treat (NNT) based on 30% and 50% reduction of pain severity ratings, as well as number-needed to harm (NNH) based on discontinuation due to adverse events (AEs) for studies 1 to 4. Additionally, for studies 1 to 3, we determined the percentage of patients in the duloxetine 60 mg QD and placebo groups with the endpoint PGI ratings ≤2 (corresponding to feeling “much better” or “very much better”) and ≤3 (“very much better”) and compared groups using the Cochran-Mantel-Haenszel test.

As noted above, it was possible to pool the data from studies 1 to 3. We used the pooled data to evaluate changes from baseline to 12-weeks in weight and glycemic control in patients receiving duloxetine 60 mg QD compared with placebo patients. For this pooled analysis, weight and glycemic control were chosen as relevant safety parameters in a patient population with DM.

## 3. Results 

### 3.1. Demographics and Baseline Assessments

Table 2 presents baseline demographic parameters and disease characteristics for studies 1 through 5.

### 3.2. Efficacy Results

#### 3.2.1. Pain-Related Outcomes

The summary of efficacy results is shown in Table 3. In studies 1 to 4, patients treated with duloxetine 60 mg QD (duloxetine 40 mg and 60 mg combined in study 4) reported statistically significantly greater reductions in pain severity ratings from baseline to 12 weeks compared with patients receiving placebo treatment (Table 3) [13–15, 18]. Mean reductions from baseline to endpoint on the BPI-Interference were significantly greater for patients receiving duloxetine compared with placebo in all 4 studies. Similarly, duloxetine groups in all studies were significantly (*P* ≤ .01) superior to placebo groups on the 12-week PGI-Improvement ratings [13–15].

Response rates (based on 30% reduction of pain severity ratings) ranged from 57% to 68% for patients receiving duloxetine compared with 35% to 47% in the placebo groups and were statistically significantly different (*P* ≤ .01) in 3 out of 4 studies. Response rates (based on 50% reduction of pain severity ratings) ranged from 39% to 50% for patients receiving duloxetine compared with 20% to 30% in the placebo groups and were statistically significantly different (*P* ≤ .05) in 2 out of 4 studies [14, 15, 18, 19]. The NNT (based on 30% pain reduction) ranged from 4.0 to 5.7. The corresponding numbers for 50% pain reduction were 4.3 to 6.5. The NNH (based on discontinuations due to AEs) ranged from 12.5 to 58.8 in studies 1 to 4. The percentage of patients with the endpoint PGI rating of ≤2 at 12 weeks ranged from 52.3% to 58.0% for patients receiving duloxetine 60 mg QD and from 29.5% to 32.4% for patients receiving placebo in studies 1 to 3 with statistically significant (*P* < .01) differences observed in all 3 studies; patients with the endpoint ≤3 PGI rating at 12 weeks ranged from 75.0% to 85.3% for patients receiving duloxetine 60 mg QD and 60.0% to 71.4% for patients receiving placebo in studies 1 to 3 with statistically significant (*P* < .01) differences observed in all 3 studies.

#### 3.2.2. Functional and Quality of Life Outcomes

Data from studies 1 to 3 were pooled by treatment group to analyze the following functional and quality of life outcome measures: BPI-Interference, SF-36, and EQ-5D [22]. Patients treated with duloxetine 60 mg QD showed significantly greater improvement, compared with placebo, in all SF-36 domains of physical functioning, role-physical, bodily pain, general health, vitality, social functioning, role-emotional, and mental health. Additionally, patients treated with duloxetine 60 mg QD reported significantly greater improvements on BPI-Interference ratings (*P* < .001) and on the EQ-5D Index (*P* = .004) compared with patients receiving placebo [22].

#### 3.2.3. Long-Term Efficacy

At the beginning of the maintenance period of study 5, a total of 115 patients (53.2% of all enrolled patients) were responders as defined by a 30% pain reduction criterion. In the responder group, 12 patients had their duloxetine doses increased during the maintenance phase and were therefore excluded from the analyses. The remaining 103 patients treated with duloxetine 60 mg QD during the entire maintenance period demonstrated a mean change in pain severity rating of 0.35, with 0.79 as the upper limit of the 97.5% CI. The prespecified noninferiority margin was 1.5—therefore, the MOE of duloxetine on pain reduction in patients with DPNP was confirmed. At the end of the study, after 34 weeks of treatment with duloxetine 60 mg QD, 66.7% of the 114 responders with a baseline and at least 1 nonmissing postbaseline BPI average pain rating had ≥50% pain reduction, generally considered substantial pain reduction [23], relative to week 0 based on BPI average pain ratings [17]. 

#### 3.2.4. Efficacy and Safety of Duloxetine 60 mg QD in Older Patients with DPNP

Data from studies 1 to 3 were pooled by treatment group and stratified by age (<65 years of age versus ≥65 years of age) [21]. In both age groups, the pain severity ratings improved significantly (*P* < .01) for patients receiving duloxetine relative to placebo. For patients ≥65 years of age, the estimated probability of a 30% reduction in pain at endpoint was 73% for duloxetine 60 mg QD and 41% for placebo; of a 50% reduction in pain at endpoint, the probability was 55% for duloxetine 60 mg QD and 27% for placebo. These probabilities of response were similar to those observed in the younger subgroup. No significant differences between age groups were observed in BPI-Interference average ratings at endpoint, and both age groups improved significantly (*P* < .05) from baseline to endpoint during treatment with duloxetine 60 mg QD compared with placebo [21].

The age subgroups did not differ significantly in the overall rates of treatment-emergent AEs (TEAEs) (*P* = .841). In patients <65 years of age, 80.8% of patients receiving duloxetine 60 mg QD and 69.1% of patients receiving placebo experienced ≥1 TEAE (*P* = .004); in patients ≥65 years of age, 81.9% of patients receiving duloxetine 60 mg QD and 70.6% of patients receiving placebo experienced ≥1 TEAE (*P* = .056). The percentages of patients who had discontinuations due to any TEAE were significantly (*P* < .001) higher in older patients, compared with younger patients, both during treatment with duloxetine 60 mg QD (16.2% versus 8.8%, resp.) and during treatment with placebo (9.2% versus 3.9%, resp.). Neither significant differences between age groups nor interaction of therapy and age was observed for any of the vital signs variables [21].

### 3.3. Tolerability and Safety Findings

#### 3.3.1. Short-Term Treatment with Duloxetine 40 mg QD or 60 mg QD

In studies 1 to 4, discontinuations due to AEs ranged from 4.3% to 14.9% of patients receiving treatment with duloxetine 40 mg QD or 60 mg QD and 2.6% to 7.4% of patients receiving placebo (Table 4). The most frequently observed TEAEs included nausea, somnolence, and headache in studies 1 to 4 (Table 4). In those studies 16 patients (0.4%) receiving duloxetine 40 mg QD or 60 mg QD and 21 patients (0.4%) receiving placebo experienced serious AEs (SAEs) (Table 5) [16, 20]. 

In study 5, 9.3% of patients receiving duloxetine 60 mg QD discontinued the initial 8-week treatment phase due to AEs. All TEAEs were analyzed from baseline to 34 weeks (acute and maintenance phase combined) in study 5 [17] and results are presented in the following section.

#### 3.3.2. Extended Treatment with Duloxetine 60 mg QD

Safety data for extended treatment with duloxetine 60 mg QD are available from study 5. Because of AEs, 14 patients (12.2%) receiving duloxetine 60 mg QD discontinued the MOE phase [24]. A total of 139 patients (64.4%) experienced at least 1 TEAE, and nausea (*n* = 41, 19.0%), somnolence (*n* = 18, 8.3%), and hyperhidrosis (*n* = 14, 6.5%) were the most frequently observed TEAEs during treatment with duloxetine 60 mg QD, acute and maintenance phase combined, although nausea was mostly reported at the initiation of treatment [24]. 

When data were pooled from all patients receiving fixed-dose duloxetine 60 mg QD in DPNP studies with extended treatment, the 9 most common AEs (nausea, somnolence, diarrhea, dizziness, fatigue, hyperhidrosis, constipation, insomnia, and dry mouth) tended to appear early during treatment with duloxetine 60 mg QD and tended to subside, with only a minority of patients having persisting events [20].

In study 5, SAEs were observed in 18 patients (8.3%) and included unstable angina, atrial fibrillation, congestive cardiac failure, hyperthyroidism, cataract, intestinal ischemia, esophageal ulcer hemorrhage, chest pain, sudden death, cholelithiasis, rib fracture, DM, diabetic ketoacidosis, hypokalemia, breast cancer, transient ischemic attack, anuria, urinary retention, dyspnoea, diabetic neuropathic ulcer, pruritus, and peripheral arterial occlusive disease [24].

#### 3.3.3. Changes in Blood Pressure and Heart Rate in Patients with DPNP during Treatment with Duloxetine 40 mg QD or 60 mg QD

No pooled analyses for changes in blood pressure and heart rate are available in the literature. In study 1, patients treated with duloxetine 60 mg QD experienced a mean increase of 2.49 bpm in heart rate and a mean increase of 2.52 mm Hg in diastolic blood pressure compared with a decrease of −0.59 bpm in heart rate and a decrease of −1.84 mm Hg in diastolic blood pressure in patients receiving placebo (*P* ≤ .05 and *P* ≤ .001, resp.) during 12 weeks of double-blind treatment. In studies 2 and 3, no statistically significant differences in changes in heart rate or blood pressure were observed between duloxetine 60 mg QD and placebo. In study 4, patients receiving duloxetine 40/60 mg QD presented mean increases in heart rate (3.2 bpm) and blood pressure (systolic: 0.7 mm Hg; diastolic: 1.4 mm Hg) compared with mean decreases in patients receiving placebo (heart rate: −2.1 bpm; systolic blood pressure: −2.1 mm Hg; diastolic blood pressure: −1.8 mm Hg) (Table 6).

#### 3.3.4. Weight Change in Patients with DPNP during Treatment with Duloxetine 60 mg QD

Data from patients from studies 1 to 3 were pooled by treatment group (placebo versus duloxetine 60 mg QD) to explore weight changes from baseline to the 12-week endpoint. Patients receiving duloxetine 60 mg QD (*n* = 332) lost significantly more weight (mean weight loss: −0.90 kg, SD = 2.91 kg; *P* < .001) compared with patients in the placebo group (*n* = 326; mean weight gain: +0.16 kg; SD = 2.62 kg). In study 4, patients receiving duloxetine 40 or 60 mg QD (*n* = 160) experienced a small mean weight loss (−0.21 kg) compared with a small mean weight gain (+0.46 kg) in patients receiving placebo (*n* = 159).

In study 5, patients receiving duloxetine 60 mg QD experienced a statistically significant (*P* ≤ .001) decrease in weight (−1.29 kg) from baseline to the 34-week endpoint [17].

#### 3.3.5. Glycemic Control in Patients with DPNP during Treatment with Duloxetine 60 mg QD

Data from patients from studies 1 to 3 were pooled by treatment group (placebo versus duloxetine 60 mg QD) to explore changes in fasting blood sugar (FBS) and glycosylated hemoglobin (HbA_1c_) from baseline to the 12-week endpoint. In patients receiving duloxetine 60 mg QD, a mean increase in FBS of +12.08 mg/dL (+0.67 mmol/L) (SD = 68.65 mg/dL [+3.81 mmol/L]) was observed, compared with a mean decrease in placebo patients (−0.36 mg/dL [−0.02 mmol/L], SD = 79.46 mg/dL [+4.41 mmol/L]). Both patients receiving duloxetine 60 mg QD and placebo patients experienced a mean decrease in HbA_1c_ (−0.14% and −0.08%, resp.).

In study 4, both patients receiving duloxetine 40 or 60 mg QD and patients receiving placebo experienced a mean increase in FBS (+8.7 mg/dL [+0.48 mmol/L] and +8.3 mg/dL [+0.46 mmol/L], resp.) and in HbA_1c_ (+0.06% and +0.10%, resp.).

## 4. Discussion

Duloxetine at doses of either 40 mg QD or 60 mg QD demonstrated efficacy in the management of DPNP based on significantly greater mean reductions in pain severity ratings and BPI-Interference ratings compared with patients receiving placebo (*P* < .05) in all fixed-dose, placebo-controlled studies [13–16, 18]. Additionally, 12-week PGI-Improvement ratings were significantly (*P* ≤ .01) superior to placebo for patients receiving duloxetine in all placebo-controlled studies [13–16]. Response rates (based on 30% and 50% pain reduction from baseline) were higher in patients treated with duloxetine compared with those treated with placebo. However, 32% to 43% of duloxetine-treated patients and 53% to 65% of placebo-treated patients did not reach 30% pain reduction from baseline in the course of treatment. In a 6-month open-label study, the MOE of duloxetine on pain reduction in patients with DPNP was confirmed [17]. The results summarized in this paper are limited to fixed-dose, placebo-controlled studies of duloxetine 40 mg QD or 60 mg QD. Additional data from studies using other duloxetine dose levels and/or flexible dosing are available in the literature. An example for a double-blind, randomized, placebo-controlled, flexible-dose study of duloxetine 60 mg QD to 120 mg QD in patients with DPNP was published by Yan and colleagues in 2010 [25]. In this study, the mean change from baseline to 12-week endpoint in BPI average pain rating was not statistically different between duloxetine-treated patients and those receiving placebo [25].

Data presented in this paper are based on a geographically diverse patient population including patients from countries in North and South America, Europe, and Asia (study 1: North and South America; study 2: USA and Puerto Rico; study 3: North America and Europe; study 4: Japan; study 5: South America and Europe). Patients in study 4 differed from patients in the other studies with regard to their race (Asian population) and lower mean body weight. Otherwise, baseline characteristics were comparable among all 5 studies [13–15, 17, 18]. Importantly, the efficacy results (as presented in Table 3) appear to be very similar among the different studies, indicating that duloxetine is efficacious in the management of DPNP in patients originating from a variety of geographic regions. Additionally, the incidence of TEAEs and the type of the observed TEAEs appeared to be comparable among the studies. Stratification of data from patients in studies 1 to 3 by age (<65 years of age versus ≥65 years of age) showed that duloxetine was overall well tolerated and efficacious for the management of DPNP in both age groups despite a higher discontinuation rate in older patients.

Duloxetine treatment was associated with a small increase in FBS (+8.7 mg/dL to +12.1 mg/dL) in the placebo-controlled studies. This phenomenon is likely related to duloxetine's noradrenergic effects since it was previously observed with other noradrenergic compounds [26]. The effect has only been observed in patients with DPNP, while it is absent in patients who receive duloxetine for other indications [12, 27]. Overall, the observed AE profile of duloxetine was consistent among studies in patients with DPNP and, with the exception of fasting glycemia, similar to profiles observed previously in patients with major depressive disorder, generalized anxiety disorder, or fibromyalgia [27]. While the observed AEs were not unexpected, they might nevertheless significantly impact individual patients who are affected. 

The interpretation of the results presented in this paper is limited by the designs of the individual studies. All studies recruited patients based on strict inclusion and exclusion criteria—patients in clinical practice often do not meet these criteria. Therefore, the results presented here might not be applicable to all patients in clinical practice. Finally, our data with regard to long-term treatment is limited to 1 open-label study including duloxetine 60 mg QD in the extension phase.

## 5. Conclusion

Duloxetine at a daily fixed dose of either 40 mg QD or 60 mg QD is efficacious in the management of DPNP. Both duloxetine doses presented safety profiles in patients with DPNP that were comparable to profiles observed in other patient populations. 

## Figures and Tables

**Table 1:** Studies of duloxetine 40 mg QD and/or 60 mg QD in the management of DPNP.

Study	Location	Number of patients	Duration and design	Treatments	Primary outcome measure
1 [13]	USA, Canada, Puerto Rico, Argentina	457	12 weeks, double blind	Duloxetine 20 mg QD	Mean change from baseline to endpoint on weekly mean of 24-hour Average Pain Severity rating
				Duloxetine 60 mg QD	
				Duloxetine 60 mg BID	
				Placebo	

2 [14]	USA, Puerto Rico	334	12 weeks, double blind	Duloxetine 60 mg QD	Mean change from baseline to endpoint on weekly mean of 24-hour Average Pain Severity rating
				Duloxetine 60 mg BID	
				Placebo	

3 [15]	Canada, Croatia, Hungary, Poland, Germany, Russian Federation	348	12 weeks, double blind	Duloxetine 60 mg QD	Mean change from baseline to endpoint on weekly mean of 24-hour Average Pain Severity rating
				Duloxetine 60 mg BID	
				Placebo	

4 [16]	Japan	338	12 weeks, double blind	40 mg QD duloxetine	Mean change from baseline to endpoint on weekly mean of 24-hour Average Pain Severity rating
				60 mg QD Duloxetine	
				Placebo	

5 [17]	Brazil, France, Germany, Italy	216	8 weeks, open label	Duloxetine 60 mg QD	NA
		184	26 weeks, open label 60 mg MOE	Duloxetine 60 mg QD	Non-inferiority test of a 1-sided 97.5% CI of the change from baseline to endpoint on the 24-hour Average Pain Severity rating
				Duloxetine 120 mg QD	

Abbreviations: BID: twice daily; CI: confidence interval; DPNP: diabetic peripheral neuropathic pain; MOE: maintenance of effect; NA: not applicable; QD: once daily.

**Table 2:** Demographics and baseline assessments in studies 1 through 5.

	Study 1 [13]		Study 2 [14]		Study 3 [15]		Study 4 [16, 18]			Study 5 [17]
	DLX 60 mg QD (*n* = 114)	PLB (*n* = 115)	DLX 60 mg QD (*n* = 114)	PLB (*n* = 108)	DLX 60 mg QD (*n* = 116)	PLB (*n* = 116)	DLX 40 mg QD (*n* = 85)	DLX 60 mg QD (*n* = 86)	PLB (*n* = 167)	DLX 60 mg QD (*n* = 216)
Mean Age, years (SD)	59.2 (11.6)	60.4 (10.5)	59.7 (11.2)	60.8 (10.6)	58.3 (10.9)	59.2 (9.8)	62.1 (9.3)	59.7 (12.1)	60.8 (9.2)	63.3 (9.5)
Female Gender, *n* (%)	35 (30.7)	56 (48.7)	40 (35.1)	39 (36.1)	68 (58.6)	63 (54.3)	20 (23.5)	24 (27.9)	38 (22.8)	104 (48.2)

					Race					

White, *n* (%)	88 (77.2)	89 (77.4)	90 (78.9)	86 (79.6)	115 (99.1)	116 (100)	0	0	0	173 (80.1)
African, *n* (%)	8 (7.0)	11 (9.6)	3 (2.6)	5 (4.6)	0	0	0	0	0	0
Hispanic, *n* (%)	13 (11.4)	12 (10.4)	16 (14.0)	17 (15.7)	0	0	0	0	0	0
Asian, *n* (%)	0	0	0	0	1 (0.9)	0	85 (100)	86 (100)	167 (100)	33 (15.3)
Other, *n* (%)	5 (4.4)	3 (2.6)	5 (4.4)	0	0	0	0	0	0	10 (4.6)
Mean weight, kg (SD)	99 (24)	94 (22)	99.9 (22.0)	104.4 (24.8)	83.3 (19.6)	87.2 (16.5)	62.7 (13.4)	65.1 (10.2)	64.5 (11.9)	84.0 (18.4)
Type 2 DM, *n* (%)	100 (87.7)	104 (90.4)	104 (91.2)	97 (89.8)	93 (80.2)	102 (87.9)	80 (94.1)	82 (95.3)	159 (95.2)	204 (94.4)
Mean duration of DM, years (SD)^a^	11.4 (8.2)	11.4 (11.3)	9.7 (9.6)	11.1 (9.1)	14.6 (8.9)	12.8 (8.6)	<5: 20 5–10: 18 ≥10: 47	<5: 16 5–10: 14 ≥10: 56	<5: 33 5–10: 32 ≥10: 97 not known: 5	14.4 (9.7)
Mean duration of DPNP, years (SD)	3.8 (4.4)	4.0 (4.1)	3.6 (3.5)	3.5 (3.2)	4.5 (4.4)	4.0 (3.5)	4.6 (3.9)	4.2 (3.7)	4.2 (4.4)	NA
Mean MNSI, score (SD)	5.1 (1.6)	5.1 (1.6)	5.5 (1.5)	5.9 (1.5)	4.9 (1.4)	5.2 (1.6)	NA	NA	NA	5.3 (1.3)
24-hour average pain severity, weekly mean (SD)	6.0 (1.7)	5.8 (1.5)	6.1 (1.6)	5.9 (1.4)	5.5 (1.1)	5.5 (1.3)	5.8 (1.2)	5.8 (1.2)	5.8 (1.2)	4.2 (4.0)

^a^For study 4, mean duration of years with diabetes was not available, displayed are the numbers of patients with a duration of diabetes of <5 years, 5–10 years, or ≥10 years for this study.

DPNP: diabetic peripheral neuropathic pain; MNSI: Michigan Neuropathy Screening Instrument; *n*: number of affected patients; NA: not available; QD: once daily; SD: standard deviation, DM: diabetes mellitus.

**Table 3:** Efficacy results of duloxetine 40 and 60 mg QD versus placebo in placebo-controlled studies in patients with DPNP for studies 1 through 4.

Measure	Study 1		Study 2		Study 3		Study 4	
	DLX 60 mg QD (*N* = 113)	PLB (*N* = 112)	DLX 60 mg QD (*N* = 112)	PLB (*N* = 106)	DLX 60 mg QD (*N* = 113)	PLB (*N* = 113)	DLX 40/60 mg QD (*N* = 171)	PLB (*n* = 167)
Weekly mean of 24 hour	−2.81**	−2.04	−2.72^∗∗∗ ^	−1.39	−2.50^∗∗∗ ^	−1.60	−2.47^∗∗∗ ^	−1.61
Average pain severity Rating, mean change (SE) [13–16]	(0.21)	(0.21)	(0.22)^b^	(0.23)	(0.18)	(0.18)	(0.18)	(0.18)
BPI-Interference, mean (SE) [13–16]	−2.33**	−1.73	−2.36*	−1.72	−2.43***	−1.56	−2.04*	−1.56
	(0.17)	(0.17)	(0.19)^a^	(0.19)^c^	(0.18)^e^	(0.18)^f^	(0.20)^i^	(0.20)^l^
PGI-Improvement, mean (SE) [13–16]	2.21^∗∗∗ ^	2.91	2.61**	3.17	2.50***	3.04	2.53**	3.18
	(0.12)^a^	(0.12)^a^	(1.44)	(1.44)^d^	(0.10)^f^	(0.10)^g^	(0.12)	(0.12)^h^
Percentage of patients with PGI ratings ≤2 at 12 weeks	57.7%^a∗∗∗^	31.5%^a^	58.0%***	32.4%^d^	52.3%^f∗∗∗^	29.5%^g^	NA	NA
Percentage of patients with PGI ratings ≤3 at 12 weeks	78.4%^a∗∗^	61.3%^a^	75.0%*	60.0%^d^	85.3%^f∗^	71.4%^g^	NA	NA
Response rate								
30% pain Reduction [14, 15]	64%^g^	47%^a^	63%**	42%	68%***	43%	57%***	35%
50% pain Reduction [14, 15, 19]	49%^g^	26%^a^	43%^b∗^	27%	50%	30%	39%***	20%
NNT Based on:								
30% pain Reduction (95% CI)	5.7 (3.3, 21.5)^g^	NA	4.7 (2.9, 12.2)^b^	NA	4.0 (2.7, 8.2)	NA	4.6 (2.7, 15.7)	NA
50% pain Reduction (95% CI) [19]	4.3 (2.8, 9.4)	NA	6.5 (3.6, 36.4)	NA	5.1 (3.1, 14.3)	NA	5.2 (2.7, 68.1)	NA
NNH based on discontinuations due to AEs (95% CI)	12.5 (6.5, 172.8)^j^	NA	13.2 (−165, 6.3)^j^	NA	58.8 (−33.4, 15.6)^k^	NA	16.0 (−74.0, 7.2)	NA

*P ≤ .05 versus placebo; **P ≤ .01 versus placebo; ***P ≤ .001 versus placebo

^a^ *n* = 111; ^b^ *n* = 110; ^c^ *n* = 104; ^d^ *n* = 105; ^e^ *n* = 108; ^f^ *n* = 109; ^g^ *n* = 112; ^h^ *n* = 165; ^i^ *n* = 85; ^j^ *n* = 114; ^k^ *n* = 116; ^l^ *n* = 165

AEs: adverse events; BPI: brief pain inventory; CI: confidence interval; DLX: duloxetine; NA: not applicable; NNH: number needed to harm; NNT: number needed to treat; PGI: patient global impression; PLB: placebo; QD: once daily.

**Table 4:** Treatment-emergent adverse events in ≥10% of patients in the duloxetine treatment arms in Studies 1 through 4.

TEAEs, *n* (%)	Study 1 [13, 20]		Study 2 [14, 20]		Study 3 [15, 20]		Study 4 [16]		
	DLX 60 mg QD (*n* = 114)	PLB (*n* = 115)	DLX 60 mg QD (*n* = 114)	PLB (*n* = 108)	DLX 60 mg QD (*n* = 116)	PLB (*n* = 116)	DLX 40 mg QD (*n* = 85)	DLX 60 mg QD (*n* = 86)	PLB (*n* = 167)
≥1 TEAE	100 (97.7)	90 (79.1)	102 (89.5)	79 (73.1)	71 (61.2)	57 (49.1)	71 (83.5)	73 (84.9)	121 (72.5)
Discontinuation due to TEAEs	15 (13.2)	6 (5.2)	17 (14.9)	8 (7.4)	5 (4.3)	3 (2.6)	9 (10.6)	10 (11.6)	9 (5.4)
Nausea	19 (16.7)	11 (9.6)	32 (28.1)	7 (6.5)	32 (27.6)	10 (8.6)	10 (11.8)	14 (16.3)	3 (1.8)
Somnolence	23 (20.2)	9 (7.8)	9 (7.9)	1 (0.9)	19 (16.4)	5 (4.3)	16 (18.8)	21 (24.4)	14 (8.4)
Headache	16 (14.0)	11 (9.6)	12 (10.5)	7 (6.5)	12 (10.3)	7 (6.0)	4 (4.7)	2 (2.3)	6 (3.6)
Dizziness	11 (9.6)	8 (7.0)	18 (15.8)	6 (5.6)	6 (5.2)	4 (3.4)	6 (7.1)	4 (4.7)	2 (1.2)
Insomnia	13 (11.4)	12 (10.4)	6 (5.3)	2 (1.9)	8 (6.9)	2 (1.7)	NA^a^	NA^a^	NA^a^
Diarrhea	11 (9.6)	11 (9.6)	13 (11.4)	2 (1.9)	8 (6.9)	6 (5.2)	4 (4.7)	7 (8.1)	6 (3.6)
Constipation	17 (14.9)	4 (3.5)	8 (7.0)	2 (1.9)	2 (1.7)	1 (0.9)	6 (7.1)	5 (5.8)	9 (5.4)

DLX: duloxetine; *n*: number of affected patients; NA: not available; PLB: placebo; QD: once daily; TEAE: treatment-emergent adverse event.

^a^Insomnia was not analyzed as a TEAE in study 4; insomnia was reported as an adverse events in 1 patient receiving placebo, 2 patients receiving duloxetine 40 mg QD, and 1 patient receiving duloxetine 60 mg QD in study 4; insomnia was reported as adverse drug reaction in 1 patient receiving duloxetine 40 mg QD in study 4.

**Table 5:** Serious adverse events in the double-blind treatment phases of studies 1 through 4.

Study *n* (%)	Patients experiencing ≥1 SAE				SAEs	
	DLX 40 mg QD (*N* = 85)	DLX 60 mg QD (*N* = 430)	PLB (*N* = 506)	DLX 40 mg QD	DLX 60 mg QD	PLB
Study 1 [20] DLX 60 mg QD (*n* = 114) PLB (*n* = 115)	NA	3 (2.6)	4 (3.5)	NA	Exhaustion; hypertension; left hip fracture; prostatic abscess; urinary tract infection; acute urinary retention	Accidental drowning; strangulated right inguinal hernia; hyperglycemia; chest pain

Study 2 [20] DLX 60 mg QD (*n* = 114) PLB (*n* = 108)	NA	4 (3.5)	7 (6.5)	NA	Congestive heart failure; coronary artery stenosis; hip fracture; prostate cancer	Heart block; fatigue; obesity; cancer to left side of the neck; COPD; diabetic ulcer; hypertension

Study 3 [20] DLX 60 mg QD (*n* = 116) PLB (*n* = 116)	NA	4 (3.5)	4 (3.5)	NA	Atrial fibrillation; cholecystitis; DM; nephrolithiasis	Anemia; cerebrovascular accident; chest pain; COPD; dyspnea; melaena; pneumonia

Study 4 [16] DLX 40 mg QD (*n* = 85) DLX 60 mg QD (*n* = 86) PLB (*n* = 167)	3 (3.5)	2 (2.3)	6 (3.6)	Alcohol poisoning; contusion; ulna fracture; hypoglycemia	Epiglottitis; radiculopathy; self injurious behavior	Abnormal hepatic function; bacterial arthritis; osteomyelitis; sepsis; tuberculous pleurisy; back injury; pathological fracture; cerebral infarction; facial palsy; hemiparesis; thalamus hemorrhage; acute renal failure

COPD: chronic obstructive pulmonary disease; DLX: duloxetine; DM: diabetes mellitus; NA: not applicable; PLB: placebo; QD: once daily; SAE: serious adverse event.

**Table 6:** LSMean changes in heart rate and blood pressure during 12-week treatment with duloxetine 40 and 60 mg QD versus placebo in placebo-controlled studies in patients with DPNP.

Measure, mean change (SD)	Study 1^a^		Study 2		Study 3		Study 4^c^	
	DLX 60 mg QD (*n* = 87)	PLB (*n* = 88)	DLX 60 mg QD (*n* = 114)	PLB (*n* = 107)	DLX 60 mg QD (*n* = 114)	PLB (*n* = 114)	DLX 40/60 mg QD (*n* = 160)	PLB (*n* = 159)
Heart rate, bpm	2.49* (0.95)	−0.59 (0.95)	−0.35^b^ (10.41)	−0.99 (9.53)	0.47 (9.02)	−0.82 (10.97)	3.2^d^	−2.1^e^
Systolic blood Pressure, mm Hg	−1.57 (1.41)	−3.50 (1.40)	−0.18 (16.33)	−0.50 (14.27)	−2.24 (14.06)	−2.89 (16.13)	0.7	−2.1
Diastolic blood Pressure, mm Hg	2.52*** (0.87)	−1.84 (0.86)	−0.29 (8.99)	−0.34 (8.82)	−0.74 (9.01)	−0.58 (9.79)	1.4	−1.8

*P ≤ .05 versus placebo; ***P ≤ .001 versus placebo

^a^Values presented are LSMean change (SE)

^b^ *n* = 113  ^c^no measure of distribution and no statistical comparison of the treatment group available

^d^ *n* = 161  ^e^ *n* = 158

bpm: beats per minute; DLX: duloxetine; LSMean: least-squares mean; *n*: number of patients; PLB: placebo; QD: once daily; SD: standard deviation; SE: standard error.
